# Jian-Pi-Yi-Shen formula improves kidney function by regulating gut microbiome in rats with chronic kidney disease

**DOI:** 10.3389/fcimb.2025.1526863

**Published:** 2025-07-09

**Authors:** Yuzhi Wang, Jiandong Lu, Wenkui Dai, Shudong Yang

**Affiliations:** ^1^ The Fourth Clinical Medical College, Guangzhou University of Chinese Medicine, Shenzhen, China; ^2^ Department of Nephrology, Shenzhen Traditional Chinese Medicine Hospital, Guangzhou University of Chinese Medicine, Shenzhen, China; ^3^ Department of Obstetrics and Gynecology, Peking University Shenzhen Hospital, Shenzhen, China

**Keywords:** traditional Chinese medicine, Jian-Pi-Yi-Shen formula, chronic kidney disease, gut microbiome, uremic toxins, metagenomic sequencing

## Abstract

**Introduction:**

Recent studies have underscored the role of interactions between Traditional Chinese Medicine (TCM) and the gut microbiome (GM) in mediating therapeutic effects. Jian-Pi-Yi-Shen Formula (JPYSF) has shown efficacy in ameliorating chronic kidney disease (CKD) symptoms, but its mechanisms via GM modulation remain unclear.

**Methods:**

In this study, 8-week-old rats were assigned to three groups after a two-week acclimation: C (normal diet for six weeks), M (adenine diet for four weeks then normal diet for two weeks), and T (same as M, with JPYSF administered during the final three weeks). Fecal samples were collected at three timepoints (T1: post-acclimation; T2: after three weeks on respective diets; T3: after three weeks of JPYSF treatment) for metagenomic sequencing. Serum creatinine (SCR) was measured at T2 and T3.

**Results:**

At T2, adenine-fed rats showed elevated SCR (C: 28.4 ± 1.5 µmol/L; M: 189.6 ± 25.8µmol/L; T: 186.4 ± 32.5µmol/L; p < 0.001). By T3, SCR decreased more in T (86.0 ± 14.9µmol/L) than in M (119.6 ± 16.3µmol/L; p = 0.012), with C remaining stable (30.8 ± 4.4µmol/L). Adenine feeding induced significant GM shifts, evidenced by increased Aitchison distance (p < 0.01) and altered co-abundance interaction groups (CIGs): CIG3, 6, 9, 10 increased; CIG1, 2, 4, 12 decreased (all p < 0.05). After JPYSF treatment, only CIG4 significantly rebounded (T3 vs. M, p = 0.0079), and T3-T1 dissimilarity was lower in T than M (p < 0.05). SCR levels were significantly lower in T than M after returning to a normal diet, suggesting a renoprotective effect of JPYSF. Co-occurrence analysis linked SCR positively with toxin-associated CIGs (CIG3, 6, 7, 9, 10) and pathways (purine metabolism, toluene degradation), and negatively with CIG4.

**Discussion:**

These results demonstrate that JPYSF lowers SCR and selectively modulates GM modules, particularly CIG4, which inversely correlates with uremic toxin–producing pathways, suggesting improved renal function and specific gut microbiota modulation in CKD rats.

## Introduction

CKD affects an estimated 434.3 million adults across Asia as of 2020 (Liyanage et al., 2022). CKD can lead to end-stage renal disease (ESRD) and associated conditions such as cardiovascular comorbidities and cachexia, resulting in nearly 1.2 million deaths annually (Bikbov et al., 2020; Kalantar-Zadeh et al., 2021). TCM has demonstrated efficacy in alleviating CKD (Mao et al., 2020; Wang et al., 2020a), yet the underlying mechanisms remain poorly understood. Recent studies highlight the role of GM dysbiosis in CKD and underscore the importance of TCM-GM interactions in disease management (Wang et al., 2020b; Zhang et al., 2022; Wang et al., 2023).

Emerging studies reported decreased levels of beneficial bacteria such as *Faecalibacterium*, *Roseburia*, *Clostridiu*m cluster IV, *Eubacterium*, *Bifidobacterium*, Lactobacillaceae, accompanied by an overrepresentation of *Enterobacteriaceae* in CKD patients (Hu et al., 2020; Wu et al., 2020; Wang et al., 2023). Wang et al. found that *Eggerthella lenta* and *Fusobacterium nucleatum* increased uremic toxin production, contributing to CKD pathogenesis (Wang et al., 2020b). Probiotic treatment with *Bifidobacterium animalis* A6 reduced these pathogenic bacteria, leading to decreased serum levels of uremic toxins, creatinine and urea, as well as attenuated glomerulosclerosis (Wang et al., 2020b). Additionally, alterations in GM and its metabolome were closely associated with serum metabolomic profiles and CKD severity (Wang et al., 2020b, 2023, 2023).

In addition, GM can impact host metabolism through interactions with diet and pharmacological agents, including TCM (Zhang et al., 2020). For example, Gegen Qinlian decoction (GQD) modulated GM and effectively alleviated type 2 diabetes (T2D) by lowering fasting blood glucose (FBG) and glycated hemoglobin (HbA1c) levels (Xu et al., 2015). Similarly, Huang-qin decoction (HQD) alleviated gastrointestinal disorders and attenuated dextran sulphate sodium-induced colitis by reducing the abundance of *Desulfovibrio* and *Helicobacter* while increasing *Lactococcus* (Yang et al., 2017). *Ganoderma lucidum* extract similarly reshaped GM by lowering the Firmicutes/Bacteroidetes ratio and reducing endotoxin-producing *Proteobacteria* in a high-fat diet-induced dysbiosis model (Chang et al., 2015). Moreover, GM is known to metabolize TCM-derived compounds, mediating therapeutic outcomes through the biotransformation of flavonoids, saponins, and anthraquinones (Bae et al., 2000, 2002; Song et al., 2011; Chiou et al., 2014; Xiao et al., 2016). While the pivotal role of GM in TCM therapy is increasingly evident, the specific interactions between TCM and GM in CKD remain to be clarified.

Jian-Pi-Yi-Shen Formula (JPYSF), a patented traditional Chinese medicine developed by Professor Li Shunmin, has been widely applied in the clinical management of CKD for decades and is regarded as a representative formula that “strengthens the spleen and kidney.” It comprises eight Chinese medicinal herbs and has shown promising efficacy and safety in CKD treatment (Liu et al., 2020a, 2021, 2022). Previous studies have demonstrated that JPYSF exerts multi-targeted effects in delaying CKD progression, including anti-inflammatory activity, improvement of iron-deficiency anemia (Li et al., 2023a), inhibition of mitochondrial fission, promotion of mitochondrial fusion, and attenuation of oxidative stress (Liu et al., 2018). In both 5/6 nephrectomy and adenine-induced CKD rat models, JPYSF has been shown to attenuate disease progression (Liu et al., 2018; Zhao et al., 2022). A recent study further demonstrated that JPYSF attenuates CKD progression by modulating macrophage polarization and amino acid metabolism, particularly through tryptophan and betaine pathways (Li et al., 2024b). Nonetheless, the precise molecular mechanisms underlying its therapeutic effects, particularly its role in modulating the gut microbiota, remain to be further elucidated.

In this study, we established the CKD rat model using a diet containing 0.75% adenine. JPYSF was subsequently administered, and its renoprotective effects were evaluated by measuring serum creatinine (SCR) levels. The adenine-induced model is a widely accepted and reproducible method for inducing CKD in rodents, and has been extensively employed to study renal pathophysiology and test therapeutic agents (Lu et al., 2023; Huang et al., 2024; Li et al., 2024b; Yang et al., 2024). GM dynamic changes in response to adenine diet and JPYSF therapies were evaluated by metagenomic analysis. Our findings provide insights into the modulation of JPYSF on GM in the treatment of CKD.

## Materials and methods

### Animal model

JPYSF is composed of eight traditional Chinese herbal medicines, including Astragalus mongholicus Bunge (Fabaceae), Atractylodes macrocephala Koidz. (Asteraceae), Dioscorea oppositifolia L. (Dioscoreaceae), Cistanche deserticola Ma (Orobanchaceae), Wurfbainia vera (Blackw), Salvia miltiorrhiza Bunge (Lamiaceae), Rheum palmatum L. (Polygonaceae), and Glycyrrhiza uralensis Fisch. ex DC. (Fabaceae). The plant names have been validated with https://mpns.science.kew.org/mpns-portal/. All raw herbs were weighed and decocted twice in 8 volumes of double-distilled water (ddH_2_O, w/v) for 1 hour each time. The decoction was centrifuged, and the supernatant was lyophilized and stored at −80°C. Before administration, the lyophilized powder was reconstituted in ddH_2_O at room temperature to obtain the JPYSF extract.

The animal experiment protocol was approved by the Ethics Committee of Shenzhen TopBiotech Co.,Ltd (TOP-IACUC-2022-0178). Fifteen male Sprague Dawley (SD) rats (8 weeks old, 200–220 g) were obtained from the Guangdong Medical Laboratory Animal Center (Guangzhou, China). Rats were housed under a 12-hour light/dark cycle (07:00–19:00 for light; 19:00–07:00 for dark) with ad libitum access to food and water, and maintained at a temperature of 22–25°C and 40–70% humidity. All experimental manipulations in this study were conducted during the light phase, specifically between 09:00 and 12:00. Each cage measured 470 mm × 312 mm × 260 mm, providing a floor area of approximately 1,466.4 cm². Three rats were housed per cage, ensuring that each animal had about 488.8 cm² of floor space, which complies with international animal welfare standards. The physical and behavioral states of the rats were monitored daily. Throughout the experimental period, no signs of abnormal behavior such as food or water refusal, aggression, self-injury, bar-biting, persistent hiding, or immobility were observed. After 14 days of acclimatization, rats were randomly divided into three groups: control group (C group, n=5), CKD model group (M group, n=5), and JPYSF treatment group (T group, n=5). CKD was induced by feeding rats an adenine-containing diet for 3 weeks. This rat model was first proposed by Yokozawa in 1986 (Yokozawa et al., 1986) and has been widely used in subsequent studies (Lu et al., 2023; Li et al., 2024b; Yang et al., 2024). The T group received JPYSF extract at 10.89 g/kg/day by oral gavage for 3 weeks. At the end of the study, all rats were anesthetized for blood collection via the abdominal aorta and euthanized by cervical dislocation under anesthesia.

### Diet composition and preparation

During the experiment, two types of diets were used: a standard pellet diet for the control group (C group), and an adenine-containing pellet diet for the CKD model group (M group) and the JPYSF treatment group (T group).

Standard pellet diet: Rats in the control group were fed a standard SPF-grade pellet diet (Guangdong Medical Laboratory Animal Center, Guangzhou, China). This diet was commercially prepared and widely used for laboratory rodents. It was supplied in cylindrical pellet form, stored at 4°C in sealed packaging, and provided ad libitum. The standard diet contained balanced amounts of protein, carbohydrates, fats, fiber, vitamins, and minerals, meeting the nutritional requirements for laboratory rats. No antibiotics or medications were added.

Adenine-containing pellet diet: The adenine powder (purity ≥99%, Sigma-Aldrich, USA) was processed into a customized adenine diet by Guangdong Medical Laboratory Animal Center. The preparation procedure was as follows: a total of 10 kg of feed containing 0.75% adenine was prepared, requiring 75 g of adenine powder. The feed was processed in two separate 5 kg batches. For each batch, 37.5 g of adenine powder was first mixed with 100 g of base diet powder to form a premix. This premix (137.5 g) was then blended with 500 g of base diet powder, and finally, the resulting mixture was combined with an additional 4362.5 g of base diet powder to yield a total of 5000 g, with a final concentration of 37.5 g/5000 g = 0.75% (w/w) adenine. The same procedure was repeated to produce the second 5 kg batch. This method ensured both precise concentration control and uniform distribution of adenine within the feed. After thorough mixing, the feed was compressed into cylindrical pellets (diameter: 12–12.5 mm; length: 30–40 mm) using a pelletizer, followed by drying at 60°C for 50 minutes. Once cooled to room temperature, the pellets were irradiated with cobalt-60 at 15 kGy for sterilization, vacuum-packed, and stored at 4°C until use. This adenine-containing diet was provided ad libitum to rats in the M and T groups for 3weeks. Daily feed intake was monitored, and blood creatinine levels were assessed after modeling to confirm successful induction of CKD.

### Sample collection and storage

Blood samples were collected from the caudal vein at T2 for all groups (C, M, and T) and from the abdominal aorta under anesthesia at T3. Serum was isolated by centrifugation and stored at -80°C. Serum creatinine (SCR) levels were measured using a creatinine assay kit (StressMarq Biosciences, British Columbia, Canada), following the manufacturer’s protocol. Fecal samples from the M and T groups were collected at T1, T2, and T3. Fecal samples were collected individually from each rat to ensure sample identity and avoid cross-contamination. Rats were housed three per cage, each identified by ear tags. During collection, one rat at a time was transferred to a clean environment. Typically, the rat would defecate within a few minutes. If not, gentle abdominal massage was applied to promote defecation. Fresh fecal pellets were collected immediately using sterile 1.5 mL Eppendorf tubes directly from the anal area, without contacting any external surfaces. Each tube was labeled and immediately placed into a liquid nitrogen tank for temporary storage. After all samples were collected, they were transferred to a –80 °C freezer for long-term storage.

### Microbial DNA extraction, sequencing and data filter

All microbial DNA was extracted from fecal samples according to the protocol of the QIAamp DNA stool kit (Qiagen, Germany). The quality of DNA was assessed via NanoDrop and Qubit (both from Thermo Fisher Scientific, Singapore). Then, the DNA fragment was selected through agarose gel electrophoresis. Metagenomic library was generated using NEBNext^®^ UltraTM DNA Library Prep Kit (NEB, USA), and sequenced by the Illumina NovaSeq 6000 platform (Illumina, United States) with 2x150bp paired-end. The high throughput sequencing data was first filtered to remove low-quality sequences, and to trim library primers and adapters using Trimmomatic (v0.39.2) with default parameters (Bolger et al., 2014). Then, the host DNA sequences were removed by mapping filtered reads against the Mus musculus genome database (C57BL/6NJ) by bowtie2 (v2.3.5.1) (Langmead and Salzberg, 2012).

### Data annotation

Sprague-Dawley rat’s fecal gene catalog was applied to annotate filtered metagenomic sequencing data (Pan et al., 2018). The abundance of taxonomy and gene function were calculated by read counts and normalized by Reads Per Kilobase per Million mapped reads (RPKM) (Pan et al., 2018).

The taxonomic classification and gene function prediction were also evaluated through other two databases. First, we chose the canonical microbe-specific gene sets from Biobakery suites (Beghini et al., 2021). The unmapped high-quality reads were classified via MetaPhlAn3 (v3.0.13), and the gene functional profiles were constructed by Humann3 against the build-in marker gene database. IGC (Integrated non-redundant Gene Catalog) containing 11M comprehensive human gut microbial genes was also applied in taxon and gene function annotation using bowtie2 (Xie et al., 2016). Then we assessed the annotation results among three databases as follows: 1) the reads mapping ratio at phylum, genus and species level, 2) the unmapped reads ratio, and 3) the gene functional annotation ratio.

### Co-abundance group and CAG-interaction group analysis

All of annotated genes were clustered into CAG based on the gene abundance profile using the Canopy algorithm with default parameters (Nielsen et al., 2014). Regard previous study and rarefaction analysis of taxon annotation and gene number, the gene clusters with more than 700 genes were defined as candidate CAGs (Nielsen et al., 2014) The abundance profiles of CAG were calculated by sample-wise median gene abundance.

The various microbes will interact with other microbes, acting as interaction groups. Therefore, the correlation distance (1-correlation coefficient) calculated by Spearman’s rank correlation was defined as the distance between different CAG. Then, we used this distance matrix and PERMANOVA (9999 permutations, P < 0.001) to cluster CAGs into GIGs (Nielsen et al., 2014; Zhang et al., 2015). The abundance of each GIG was calculated as the sum of the abundance of all the microbial CAGs in the GIG (Nielsen et al., 2014; Zhang et al., 2015).

### Classification of CAG in human GM reference gene catalogue and public database

The CAG was supposed to represent one kind of metagenomic species. The taxonomical classification of all the genes from CAGs was annotated by mapping genes to human GM reference gene catalogue. We also conducted the gene sequence similarity analysis against the NCBI RefSeq database using BLASTN (e-value <1e-5), and selected the best hit as taxonomical classification. If more than 50% of the genes from one CAG were assigned a taxon, and 90% of taxonomical genes were mapped to the same taxon, then the CAGs could be defined as one species (Zhang et al., 2015).

### Statistics and visualization

Based on unweighted UniFrac, principal coordinates analysis (PCoA) was performed. Given the relatively small sample size and the presence of potential non-normal distributions, nonparametric tests were used. Specifically, the Wilcoxon rank-sum test was employed to evaluate inter-group differences, while the Wilcoxon signed-rank test was applied to assess intra-group differences across time points for the same animals. Spearman coefficient was used to evaluate the correlation between CIG as well as between CIG and GM functional pathways and SCR level. Aitchison distance was calculated by R package coda.base. It represents the Euclidean distance after clr transformation, assessing the dissimilarity of two samples according to microbial compositions. Analysis results were visualized using R software (version 4.0.5).

## Results

### Study design

**Figure 1:**
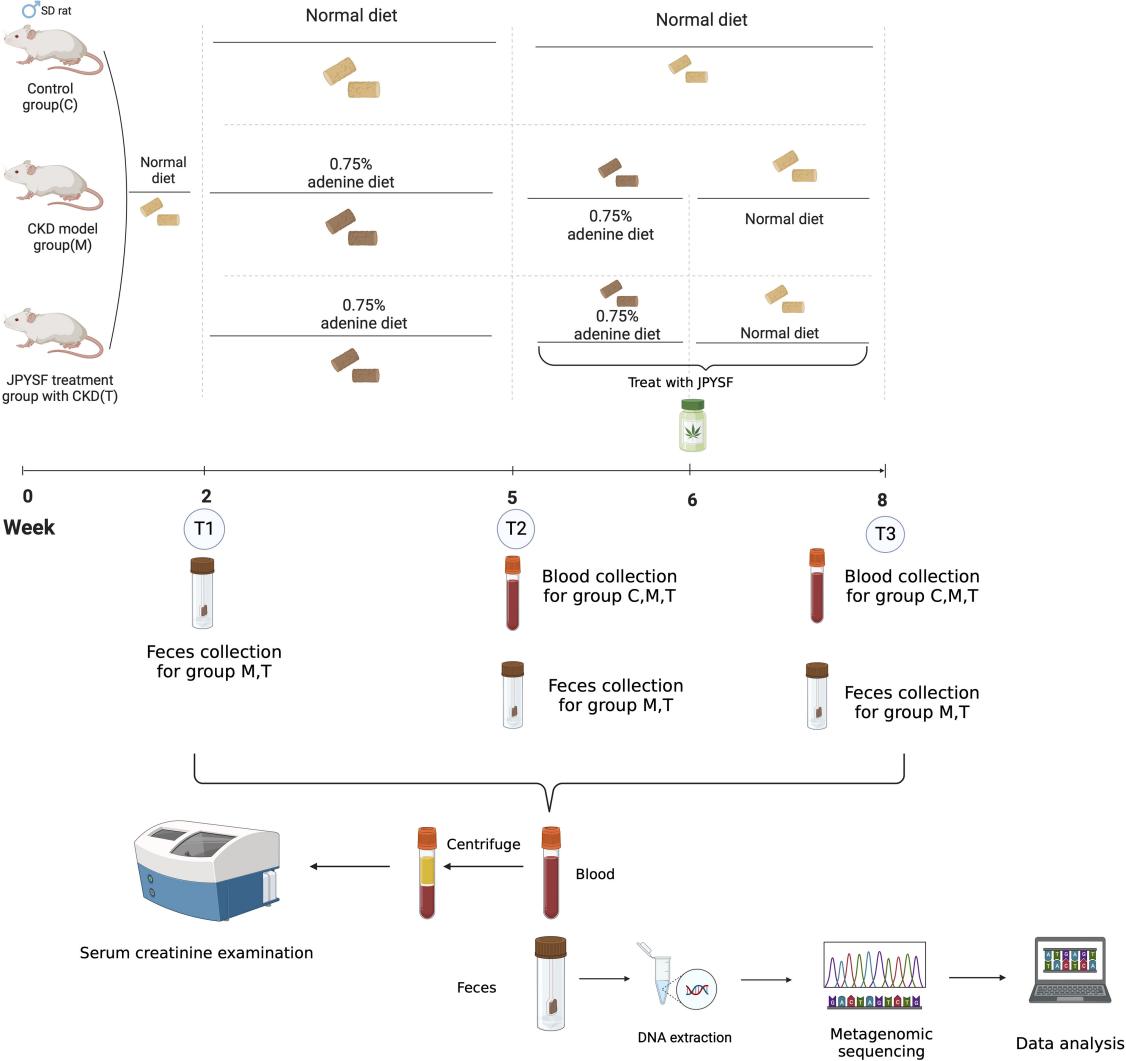
Study design and workflow. This figure applied icons in BioRender.com.

Utilizing the adenine-induced renal failure mouse model, we evaluated the effects of JPYSF on CKD and the associated changes in the gut microbiome (GM) under adenine diet and JPYSF intervention (**Figure 1**). We analyzed SCR levels in C and T groups at timepoint T2 to evaluate the detrimental effects of the adenine diet on kidney function (**Figure 1**). Additionally, we explored GM dynamic changes in response to the adenine diet (**Figure 1**). Furthermore, SCR levels at timepoint T3 were compared between the M and T groups to assess the therapeutic efficacy of JPYSF in CKD (**Figure 1**).

### Taxonomic annotation of metagenomics data

The size of clean reads ranged from 3.67 to 8.45 Gb. Approximately 45.16% of clean reads could be annotated at the phylum level when mapped to the rat reference gene catalogue, while 21.75% and 4.58% could be annotated at the genus and species levels, respectively. In contrast, only 6.17% of clean reads could be annotated at the phylum level using MetaPhlAn, with 4.55% and 4.58% at the genus and species levels, respectively. Moreover, only 6.98%, 5.18%, and 2.66% of clean reads could be assigned to the human reference gut gene catalogue at the phylum, genus, and species levels, respectively.

### SCR level and GM significantly changed after feeding adenine diet

**Figure 2:**
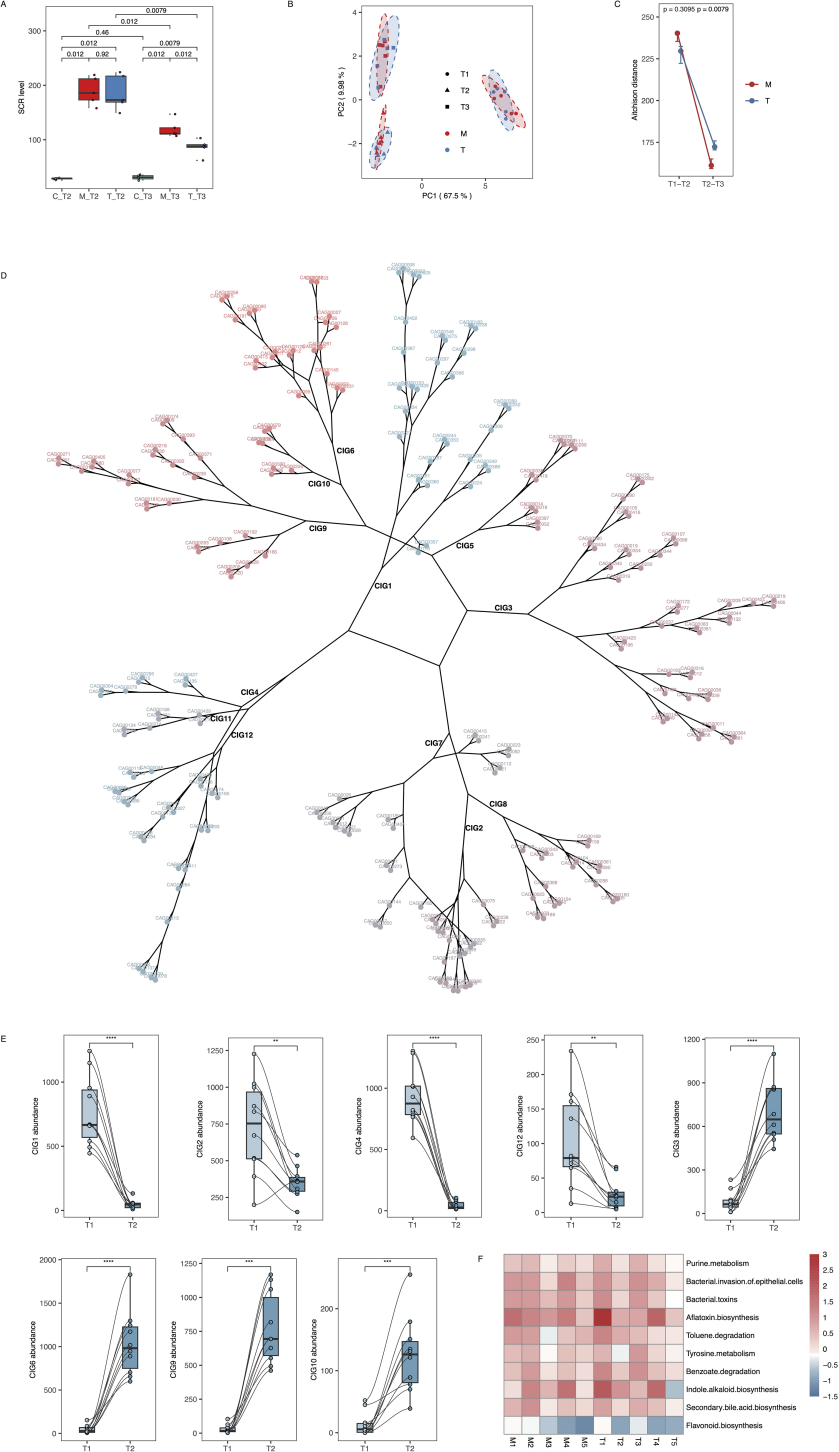
Variations in SCR levels and GM composition following adenine diet modeling. **(A)** SCR level in C, M and T groups at T2 and T3. The number up the boxplots represents p value based on Wilcoxon rank-sum test. **(B)** PCoA analysis based on unweighted UniFrac for all microbial samples. **(C)** The Aitchison distance of microbial samples between T1 and T2, as well as T2 and T3 in M and T groups. The p-values above the points indicate comparisons between M and T groups at corresponding time points, calculated using the Wilcoxon rank-sum test. **(D)** CAG components in each CIG. The cluster tree were constructed based on Aitchison distance. **(E)** Comparison of the relative abundance of selected CIGs between T1 (baseline) and T2 (post-modeling) in 10 rats from both M and T groups. This figure highlights the immediate impact of the high-adenine diet on GM composition. **, *** and **** represent p value ≤0.01, 0.001, 0.0001, respectively. **(F)** The heatmap of several GM functional pathways. The color represents log2 value of T2/T1 ratio. M1–5 represent rats in the M group, and T1–5 represent rats in the T group.

Following the adenine diet, rats in the M and T groups exhibited significantly elevated SCR levels compared to rats in the C group (T2: C group 28.4 ± 1.517 umol/L, M group 189.6 ± 25.75 umol/L, T group 186.4 ± 32.52 umol/L) (**Figure 2A**, **Supplementary Table 1**). Additionally, as measured by Aitchison distance, notable variations in GM were observed after kidney impairment (**Figure 2B**). Between T2 and T1, both the M and T groups showed high Aitchison distances, indicating that the adenine diet induced significant microbiota shifts compared to baseline. There was no significant difference between the two groups at this stage (p > 0.05), suggesting that the CKD model itself caused comparable GM changes in both (**Figure 2C**). Among the 12 co-abundance groups (CIGs) for GM (**Figure 2D**; **Supplementary Table 2**), the levels of CIG3, 6, 9, and 10 significantly increased, while the levels of CIG1, 4, 12, and 2 significantly decreased after modeling (**Figure 2E**; **Supplementary Figure 1A**, **Supplementary Table 3**). These changes were consistently observed in both M and T groups, reflecting the robust impact of the high-adenine diet on GM structure during disease induction. Further analysis revealed altered levels of GM functional pathways post-modeling, including elevated levels of purine metabolism and toluene degradation, as well as reduced levels of flavonoid biosynthesis (**Figure 2F**; **Supplementary Figure 1B**, **Supplementary Tables 4**, **5**). In the model group (M), all three alpha-diversity metrics—Chao1 richness, Shannon diversity, and Simpson diversity—exhibited a progressive increase from T1 through T3, reflecting a continual shift in gut microbial composition during CKD induction. In contrast, the treatment group (T) showed elevated Chao1, Shannon, and Simpson indices at T2 relative to T1, but following three weeks of JPYSF administration (T3), the upward trajectories of all three metrics were markedly attenuated. This suppression of diversity increases by JPYSF suggests that the formula can mitigate CKD-associated dysbiosis, maintaining microbial richness and evenness closer to pre-modeling levels (**Supplementary Figures 3A–C**).

### JPYSF significantly reduced SCR levels and restored specific CIGs in the treatment group compared to the model group

After two weeks on a normal diet (T3), both M and T groups had elevated SCR levels relative to the C group (M vs. C: p = 0.012; T vs. C: p = 0.0079), but the T group showed significantly lower SCR than the M group (T = 86.0 ± 14.9 µmol/L vs. M = 119.6 ± 16.3 µmol/L; p = 0.012; **Figure 2A**). In the T3 vs. T2 comparison, Aitchison distances decreased in both groups, reflecting some level of microbiota recovery. However, the T group displayed significantly higher Aitchison distances than the M group (p = 0.0079), indicating that the gut microbiota in the T group underwent more substantial changes after JPYSF treatment than the M group did under normal diet alone. This suggests that JPYSF exerted a greater regulatory effect on gut microbiota composition than spontaneous recovery (**Figure 2C**). GM structural shifts persisted, but samples from the T group displayed reduced Aitchison dissimilarity between T3 and T1 compared to the M group (**Supplementary Figure 1C**).

**Figure 3:**
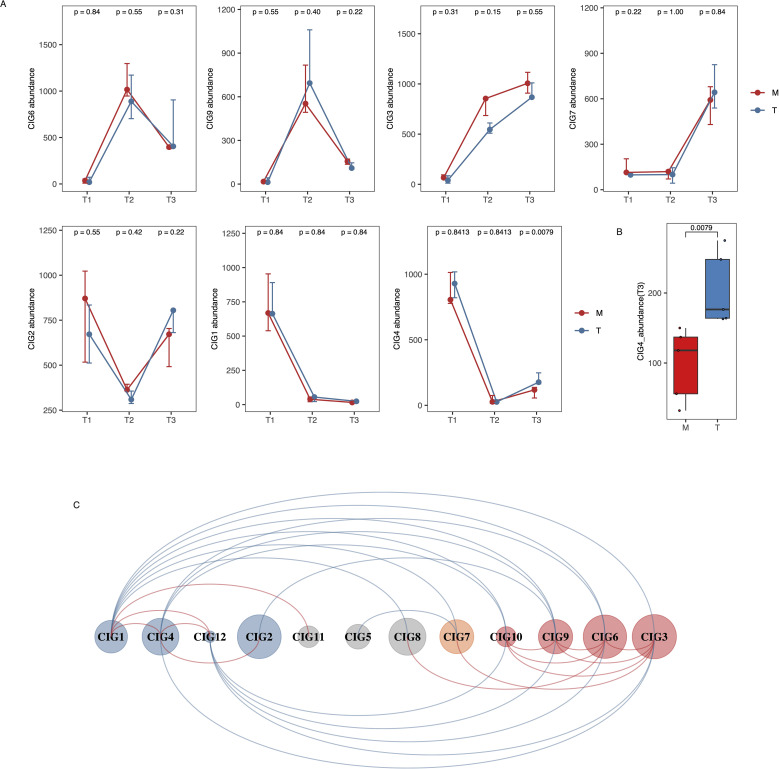
Longitudinal analysis of CIG dynamics in M and T groups across all time points. **(A)** Dynamic changes in selected co-abundance interaction groups (CIGs) from T1 (baseline), T2 (after adenine-induced modeling), to T3 (after intervention phase), showing how gut microbiota composition evolved in M and T groups. The M group resumed a normal diet after T2, while the T group received JPYSF treatment. This figure captures both the impact of CKD modeling (T1 to T2) and subsequent group-specific changes during the recovery phase (T2 to T3). The p-values above the points indicate comparisons between M and T groups at each time point, calculated using the Wilcoxon rank-sum test. **(B)** Different levels of CIG4 between M and T group at T3. The number up the boxplots represents p value based on Wilcoxon rans-sum test. **(C)** Co-occurrence network of 12 CIGs. Red and blue lines represent positive and negative correlation, respectively.

To further understand the variations in GM alterations between the M and T groups, we analyzed the dynamics of 12 CIGs across the three timepoints. Among the eight CIGs that showed notable changes after modeling, CIG2, 6, and 9 recovered to pre-modeling levels in both the M and T groups (**Figure 3A**). In contrast, CIG1 and 4 maintained low levels, while the level of CIG3 continued to increase in both groups (**Figure 3A**). Notably, a significant difference was observed in the post-treatment level (T3) of CIG4 between the M and T groups (p=0.0079) (**Figures 3A, B**). Surprisingly, the level of CIG7, which was low before and after modeling, increased significantly after treatment in both groups (**Figure 3A**). These findings from **Figure 3A** emphasize the dynamic recovery trajectories of specific CIGs over time and demonstrate distinct responses to JPYSF intervention compared to spontaneous recovery. For the post-treatment levels of other CIGs, a significant difference between the M and T groups was only observed for CIG11 (**Supplementary Figures 2A, B**).

Further analysis revealed positive correlations among CIGs that exhibited similar changes after modeling (**Figure 3C**). For instance, CIG4 positively correlated with CIG1, 2, and 12, while CIG3 positively correlated with CIG6, 7, 9, and 10 (**Figure 3C**). Conversely, CIGs whose levels increased after modeling negatively correlated with those whose levels decreased significantly after modeling (**Figure 3C**).

### T group-accumulated CIGs had negative association with SCR level

**Figure 4:**
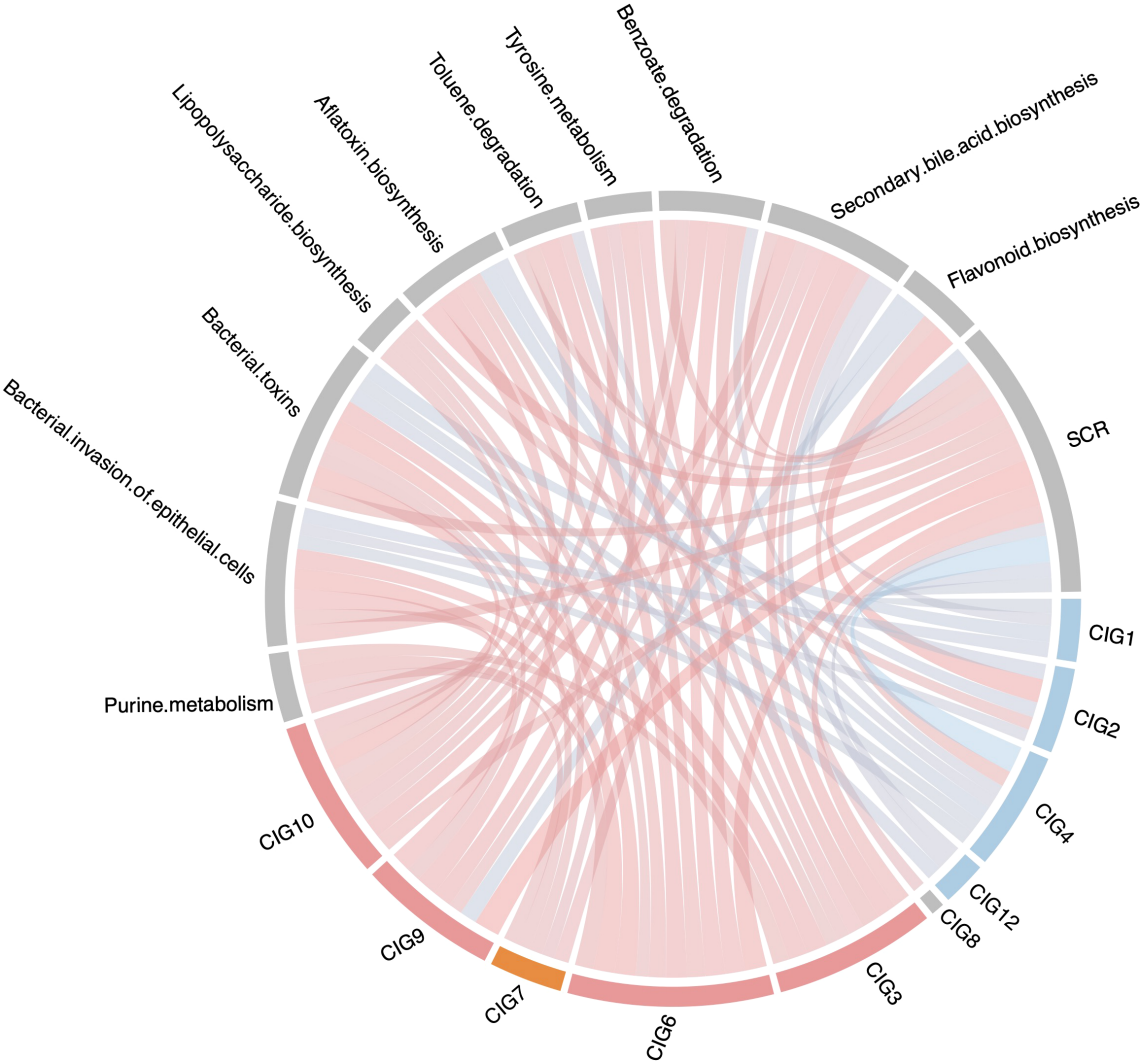
The correlation between CIGs, GM functional pathways and SCR level. The connection line represents correlation: red color means positive correlation and blue color means negative correlation; color depth and line width represents the value of spearman correlation coefficient.

To investigate the association of CIGs with kidney function, we conducted co-occurrence analysis for CIGs, GM functional pathways, and SCR levels. We observed positive associations between SCR level and CIGs whose levels significantly increased after feeding an adenine diet, such as CIG3, CIG6, and CIG9 (**Figures 3A**, **4**). Additionally, CIG7 also positively correlated with SCR level (**Figure 4**). In contrast, SCR level negatively correlated with CIGs whose levels decreased considerably after modeling, such as CIG4 (**Figures 3A**, **4**). As shown in **Figure 3A**, the abundance of CIG4 was significantly higher in the T group than in the M group at T3 (p = 0.0079). Correlation analysis further revealed that CIG4 is inversely correlated with serum creatinine (SCR) and nephrotoxin–associated pathways (e.g., aflatoxin biosynthesis, bacterial toxins, bacterial invasion of epithelial cells), while it positively correlates with flavonoid biosynthesis—a pathway known to support microbial homeostasis, protect the intestinal barrier, and exert antioxidative and anti-inflammatory effects in the kidney (**Figure 4**). Although the precise taxonomic identity of CIG4 remains to be determined, its high baseline abundance at T1, decline at T2 following adenine feeding, and significant recovery in the T group at T3 (compared to a partial recovery in the M group) support a potential renoprotective role. We also noted similar, albeit non-significant, trends for CIG2 and CIG9 at T3 (p = 0.22 for both).

Furthermore, CIG3, 6, 7, 9, and 10 positively correlated with functional pathways such as purine metabolism and benzoate degradation—both positively associated with SCR—while these pathways negatively correlated with CIG4 (**Figure 4**). These findings suggest that JPYSF not only significantly reduced SCR levels but also restored specific CIGs in the treatment group compared to the model group, potentially contributing to improved kidney function.

## Discussion

TCM is increasingly utilized in the treatment of CKD, a significant health risk for millions of individuals (Liyanage et al., 2022). However, the underlying mechanisms of TCM therapy remain largely unexplored. Growing evidence supports the importance of TCM–GM interactions in mediating therapeutic benefits (Bae et al., 2000, 2002; Song et al., 2011; Chiou et al., 2014; Chang et al., 2015; Xu et al., 2015; Xiao et al., 2016; Yang et al., 2017; Zhang et al., 2022). Indeed, CKD is now recognized to feature a marked loss of microbial richness and α-diversity that worsens as kidney function declines, with peritoneal dialysis patients showing particularly low diversity alongside expansion of Proteobacteria such as Enterobacteriaceae and Escherichia-Shigella (Li et al., 2023b; Shi et al., 2023) In this study, we employed a CKD rat model to evaluate the efficacy of JPYSF in improving kidney function and to analyze GM variations in response to JPYSF treatment.

The adenine-induced chronic kidney disease (CKD) model is a widely used and well-established non-surgical model that replicates many of the hallmarks of human CKD (Yang et al., 2024). Upon ingestion at non-physiological doses, adenine is metabolized to 2,8-dihydroxyadenine (2,8-DHA), a poorly soluble compound that crystallizes and accumulates in the renal proximal tubules. These crystal deposits cause tubular obstruction, epithelial cell damage, and trigger persistent inflammation and fibrosis. Pathophysiological changes include increased serum creatinine and blood urea nitrogen levels, proteinuria, tubular dilatation, epithelial detachment, and interstitial fibrosis. The development of renal injury involves several interconnected mechanisms. First, 2,8-DHA crystal accumulation activates innate immune pathways (e.g., TLRs, NF-κB) and induces chronic inflammation, marked by macrophage infiltration and cytokine release (Klinkhammer et al., 2020). Second, oxidative stress, exacerbated by tubular obstruction and hypoxia, leads to increased ROS production and mitochondrial dysfunction (Ullah et al., 2019). Third, various forms of programmed cell death—including ferroptosis, autophagy, and necroptosis—are observed in renal epithelial cells (Belavgeni et al., 2020; Tang et al., 2020; Carney, 2021). Fourth, extensive metabolic disturbances, particularly in tryptophan, bile acid, and phospholipid metabolism, contribute to the pathogenesis and progression of renal damage (Kobayashi et al., 2014; Miao et al., 2022). Finally, fibroblast-to-myofibroblast transition is driven by pro-fibrotic signaling pathways, such as TGF-β/Smad and Wnt/β-catenin, resulting in excessive extracellular matrix deposition and tubulointerstitial fibrosis (Yi et al., 2021; Wang et al., 2022b). Collectively, these mechanisms mirror key features of human CKD, including progressive loss of renal function, inflammation, oxidative stress, and fibrosis, making the adenine model a robust and reproducible tool for studying CKD pathogenesis and therapeutic interventions.

As expected, SCR increased significantly following adenine feeding (Diwan et al., 2018). Notably, GM variations following modeling were in line with previous findings, indicating the impact of diet on GM (Rothschild et al., 2018; Zhao et al., 2018; Bolte et al., 2021). For instance, Zhao et al. found that a high-fiber diet selectively promoted GM producers of short-chain fatty acids, thereby alleviating type 2 diabetes (Zhao et al., 2018). Given that SCFAs such as acetate, propionate, and butyrate are significantly depleted in CKD and DKD patients—and these decreases correlate with renal dysfunction and inflammation—the ability of TCM formulas (e.g., Shenyan Kangfu, Tangshen Formula, QiDiTangShen) to restore SCFA levels and engage GPR41/43 to suppress NF-κB signaling may underlie their anti-fibrotic effects (Mao et al., 2023; Zhao et al., 2023, 2024). Population and animal studies have also reported GM changes in response to an adenine diet (Zhu et al., 2018; Liu et al., 2020b), which was used to induce CKD-like symptoms in rats in our study. However, we could not identify known CKD GM biomarkers in our model rats (Hu et al., 2020; Wu et al., 2020; Zhang et al., 2022; Wang et al., 2023), likely due to the limited amount of sequencing data that could be assigned to known taxonomic units.

Although many GM components in our rat models remained unknown, we identified the accumulation of several GM functional pathways following modeling. These accumulated functional pathways were associated with the production of toxins that impair kidney function. For example, p-cresol, a product of toluene degradation, has been shown to have dramatically elevated serum levels in CKD patients (Salmean et al., 2015; Wang et al., 2020b). Additionally, the products of tyrosine and phenylalanine metabolism, phenol and phenylacetylglutamine, are toxic to the kidney and are increased in CKD patients (Coan et al., 1982; Adeyemi et al., 2009; Posada-Ayala et al., 2014; Poesen et al., 2016; Wang et al., 2020b, 2023). Previous studies have observed compromised intestinal barriers in CKD patients, facilitating the translocation of these GM-derived uremic toxins into the bloodstream (Yang et al., 2019). These predicted shifts mirror reports that CKD features down-regulated tryptophan metabolism and SCFA biosynthesis alongside up-regulated oxidative-stress and uremic-toxin modules, driving accumulation of indoxyl sulfate, phenyl sulfate, and indole-3-acetic acid to exacerbate renal fibrosis (Li et al., 2024a). This may explain the accumulation of GM functional pathways related to bacterial invasion of epithelial cells in our study.

After switching to a normal diet for two weeks, SCR levels decreased in both the M and T groups, with a significantly greater reduction observed in the T group, and GM variations were observed in all rats. This is consistent with prior research indicating that diet can impact kidney health through modulation of the GM (Kieffer et al., 2016; Lai et al., 2019) Notably, rats receiving JPYSF treatment had significantly reduced SCR levels compared to those not receiving treatment. This may be partially explained by the regulation of TCM on the intestinal barrier and microbial flora in CKD treatment (Wang et al., 2022a; Xie et al., 2022). Consistently, we observed the selective promotion of GM CIG4 following JPYSF therapy. Additionally, GM can metabolize TCM components to produce specific bioactive molecules beneficial to host health (Bae et al., 2000, 2002; Song et al., 2011; Chiou et al., 2014; Xiao et al., 2016). Crucially, targeted probiotic approaches recapitulate these effects: in adenine-CKD rats, loss of Lactobacillus johnsonii correlates with renal deterioration, while its supplementation restores fecal L. johnsonii, boosts indole-3-aldehyde—and protects via AhR suppression; in PD patients, L. paracasei N1115 shifts GM toward Firmicutes and relieves GI symptoms—pointing to microbiota as both marker and mediator of TCM efficacy (Miao et al., 2024a; Zhou et al., 2024). Membranous nephropathy (MN) models and IMN patients share a distinct depletion of five probiotics—L. johnsonii, L. murinus, L. vaginalis, L. reuteri and Bifidobacterium animalis—which correlates with altered tryptophan-indole profiles and up-regulated intrarenal AhR signaling (Miao et al., 2024b).

Fecal samples primarily represent the luminal gut microbiota, which may not fully reflect the mucosa-associated microbial communities that interact more directly with the host, particularly at the intestinal epithelium and immune interface. In our study, fecal sampling was chosen due to its non-invasive nature and practicality in tracking microbiota changes longitudinally at multiple time points (T1, T2, and T3) without sacrificing animals. This allowed us to monitor microbial dynamics throughout disease progression and treatment. Moreover, previous studies have shown that although fecal and mucosal microbiota differ in composition, fecal samples still provide valuable insights into global shifts in microbial diversity and metabolic potential, especially when combined with metagenomic analysis.

Indeed, recent studies have highlighted the compositional and functional differences between mucosa-associated and fecal microbiota. For example, a shotgun metagenomic analysis of matched rectal mucosa and feces from patients with colonic polyps revealed that mucosal samples contained fewer genera and exhibited distinct enrichment in pathways related to sugar transport and short-chain fatty acid metabolism, underscoring the complexity and tissue-specificity of the gut microbiota (Yin et al., 2024). Insights from animal studies show distinct differences between fecal and mucosal microbiota. For example, a rat study comparing small intestine and feces found that Escherichia−Shigella, Lactobacillus, Romboutsia, Rothia, Streptococcus, and Turicibacter were dominant in ileal contents but were dramatically reduced in fecal samples, whereas genera like Lachnospiraceae, Muribaculaceae, Akkermansia, and Ruminococcaceae were much more prevalent in feces (Sun et al., 2024). Additionally, another mouse study demonstrated that microbiota diversity and taxa richness in the entire gastrointestinal tract were significantly higher than those in feces, particularly at finer taxonomic levels (Tanca et al., 2017). Therefore, if we had analyzed mucosa-associated or luminal intestinal extracts, we would expect:

1. Additional taxa detected—especially genera closely associated with the mucosal surface or upper small intestine (e.g., Escherichia−Shigella, Lactobacillus, Turicibacter, Romboutsia) that may have been underrepresented or absent in fecal samples.
2. Higher overall diversity and richness, particularly at the genus and species levels, reflecting more complex ecological niches than those captured in feces.
3. Differences in functional profiles, such as increased detection of metabolic pathways associated with epithelial interactions, mucosal immunity, or nutrient absorption processes.

In future work, we plan to integrate both fecal and intestinal mucosal samples to gain a more comprehensive understanding of how JPYSF modulates the gut ecosystem, including host–microbe interactions at the mucosal interface.

Our study observed notable GM variations in response to diet and JPYSF therapy, and the association of these variations with SCR levels. However, several limitations should be noted. First, the changed GM CAGs and CIGs remained unknown based on annotation using rat GM reference gene catalogues and public databases. Furthermore, these CAGs and CIGs were not found in the human GM reference gene catalogue, suggesting differences in GM structures and responses to diet and TCM between rats and humans. This emphasizes the importance of TCM interventions in animals receiving fecal microbiota transplantation (FMT) from host donors. Second, the mediation of GM in JPYSF therapy could not be assessed via association analysis alone in our study. Future studies should include antibiotics-treated or germ-free rats to investigate the role of GM in JPYSF treatment. Third, only SCR was used to assess kidney function. Other indicators and pathological testing, such as serum urea nitrogen, cystatin C level, urinary albumin analysis, should be considered. The lack of renal histological data restricts our ability to draw conclusions about tissue-level improvements. Although histological analysis was not feasible due to internal data constraints, previous studies using the same JPYSF formula in adenine-induced CKD rat models have demonstrated significant improvements in renal pathology following treatment (Liu et al., 2020a, 2022; Li et al., 2024b). Our study focused primarily on the dynamic changes in gut microbiota composition and serum creatinine levels, which serve as important but indirect indicators of kidney status. Future studies will incorporate histological evaluation to further validate these findings. Additionally, renal function was not assessed before feeding the adenine diet in the M and T groups. However, to evaluate the changes in SCR levels in the M and T groups, which indicated impaired kidney function after switching to an adenine diet, we included rats fed normal diets as controls. Fourth, we did not perform GM analysis for control rats. We focused our resources on a within-subject longitudinal design, in which each animal serves as its own baseline at T1. This should not negatively impact our conclusions, as our aim was to understand how GM dynamics changed in response to the adenine diet and JPYSF treatment. In summary, this study demonstrated adenine diet-induced kidney impairment and GM variations. JPYSF therapy was found to lower SCR levels and selectively modulate GM structures. Moreover, TCM’s renoprotective effects align with a growing body of evidence showing that herbal formulas restore SCFA- and indole-based metabolite profiles, correct amino-acid–derived uremic toxins, and attenuate oxidative-stress and inflammatory signaling across diabetic and non-diabetic CKD models—validating the gut–kidney axis as a key therapeutic target (Mao et al., 2023; Zhao et al., 2023; Yu et al., 2024; Zhao et al., 2024). Our findings contribute to a deeper understanding of TCM therapy in CKD from the perspective of GM interactions.

## Data Availability

Sequence data that support the findings of this study have been deposited in the CNGB Sequence Archive (CNSA) under accession number CNP0004120 and are available at the following URL: https://db.cngb.org/search/project/CNP0004120/.
