# Supplementary material for: Jian-Pi-Yi-Shen formula improves kidney function by regulating gut microbiome in rats with chronic kidney disease
Source: Front Cell Infect Microbiol. 2025 Jul 9;15:1526863. doi: 10.3389/fcimb.2025.1526863 (PMC12283701; doi:10.3389/fcimb.2025.1526863)
Supplement: Supplementary file 6 [file DataSheet6.pdf]

Supplementary Fig. 1. Variations of GM in reaction to modeling via high-adenine diets. (A) Relative abundance of several CIGs at T1 and T2 for 10 rats. (B) Relative abundance of several GM functional pathways at T1 and T2 for 10 rats. *, ** and *** represent p value <0.05, 0.01, 0.001, respectively. Light blue represents 10 rats before modeling (T1) while dark blue represent post-modeling (T2). (C) The Aitchison distance of microbial samples between T1 and T2, as well as T2 and T3 in M and T groups. This assessed whether rats in those two groups had similar GM dynamics.

Supplementary Fig. 2. CIG variations in M (red color) and T groups (blue color). (A) Dynamic changes of several CIGs following diet change and TCM treatment. The number up the points represent p value based on Wilcoxon rans-sum test. (B) Different levels of several CIGs between M and T group at T3. The number up the boxplots represent p value based on Wilcoxon rans-sum test.

Supplementary Fig. 3. JPYSF treatment partially restores gut microbial-diversity in adenine-induced CKD rats. (A) Chao1 richness index. (B) Shannon diversity index. (C) Simpson diversity index.

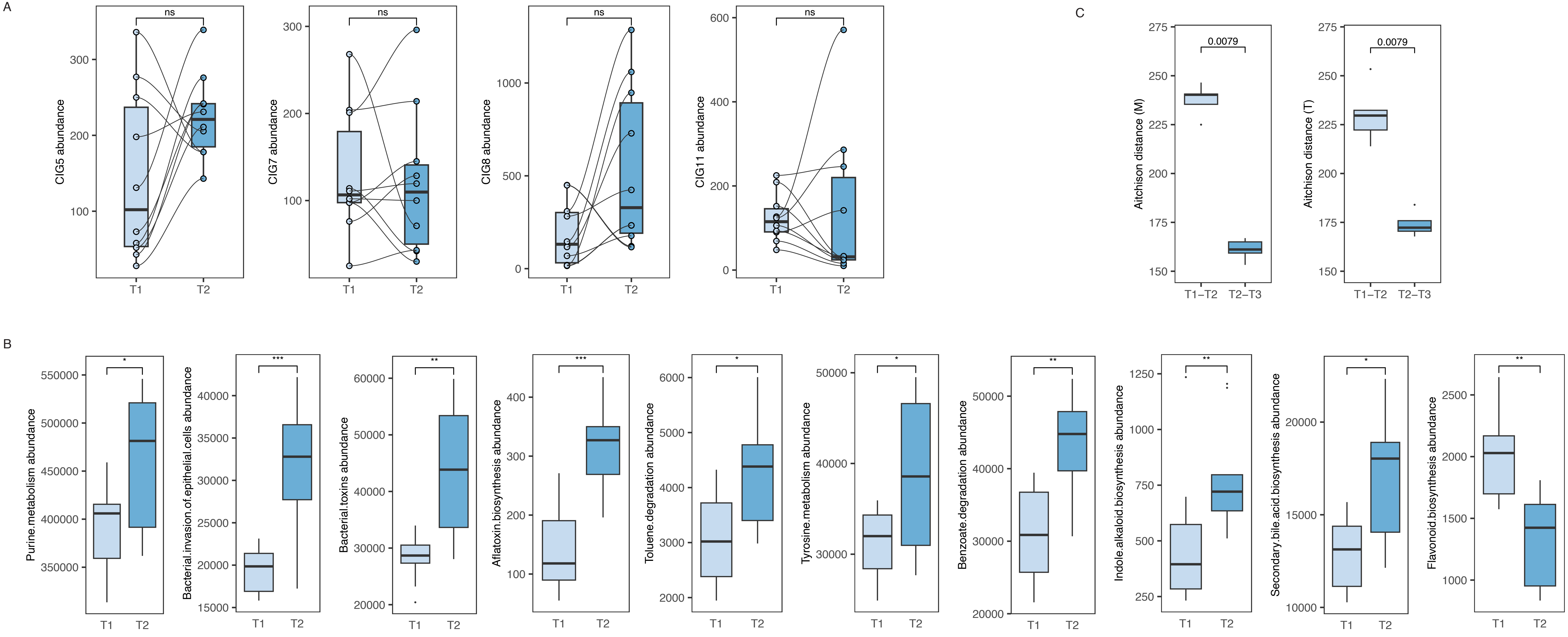

Supplementary Fig. 1

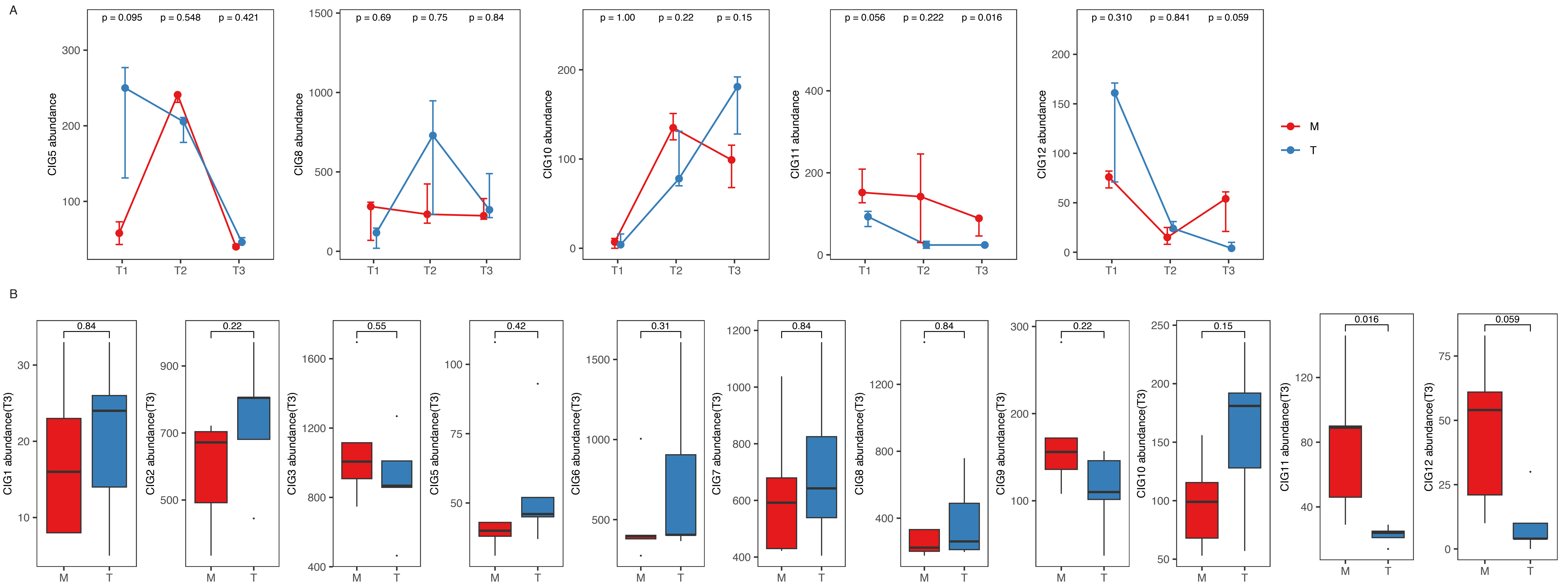

Supplementary Fig. 2

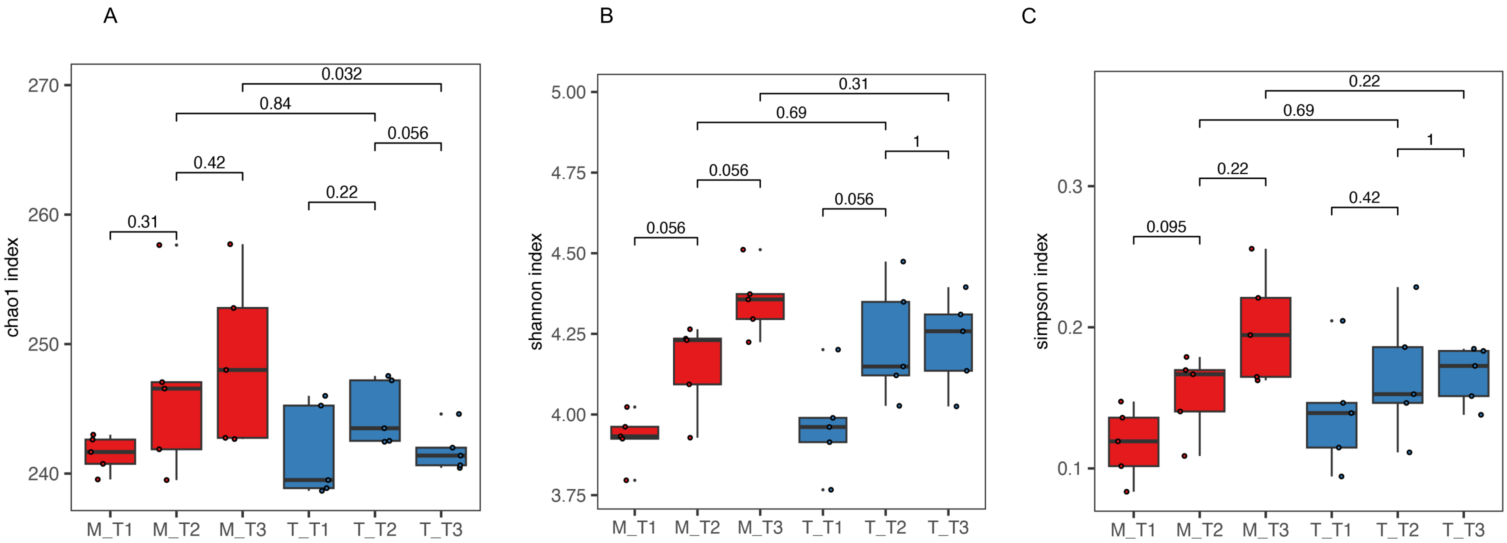

Supplementary Fig. 3
